# Hydrogen-Chlorate Electric Power Source: Feasibility of the Device, Discharge Characteristics and Modes of Operation

**DOI:** 10.3390/molecules27175638

**Published:** 2022-09-01

**Authors:** Dmitry V. Konev, Olga I. Istakova, Evgeny A. Ruban, Artem T. Glazkov, Mikhail A. Vorotyntsev

**Affiliations:** 1 Frumkin Institute of Physical Chemistry and Electrochemistry, Russian Academy of Sciences, Moscow 119071, Russia; 2 Institute for Problems of Chemical Physics, Russian Academy of Sciences, Chernogolovka 142432, Russia

**Keywords:** hybrid power source, chlorate electroreduction, hydrogen anode, mediator autocatalysis, high specific energy storage

## Abstract

A power source based on the current-generating reaction of aqueous chlorate-to-chloride reduction by molecular hydrogen would provide as much as 1150 Wh per 1 L of reagent storage (for a combination of 700 atm compressed hydrogen and saturated aqueous solution of lithium chlorate) at room temperature, but direct electroreduction of chlorate only proceeds with unacceptably high overvoltages, even for the most catalytically active electrodes. In the present study, we experimentally demonstrated that this process can be performed via redox-mediator catalysis by intermediate products of chlorate reduction, owing to their participation in homogeneous com- and disproportionation reactions. A series of current–voltage and discharge characteristics were measured for hydrogen-chlorate membrane–electrode assembly (MEA) cells at various concentrations of chlorate and sulfuric acid under operando spectrophotometric monitoring of the electrolyte composition during the discharge. We established that chlorine dioxide (ClO_2_) is the key intermediate product; its fraction in the electrolyte solution increases progressively, up to its maximum, equal to 0.4–0.6 of the initial amount of chlorate anions, whereas the ClO_2_ amount decreases gradually to a zero value in the later stage. In most discharge experiments, the Faradaic yield exceeded 90% (maximal value: 99%), providing approximately 48% chemical energy storage-to-electricity conversion efficiency at maximal power of the discharge (max value: 402 mW/cm^2^). These results support prospect of a hydrogen-chlorate flow current generator as a highly specific energy-capacity source for airless media.

## 1. Introduction

The area of inhabitancy and economic activity of mankind is steadily expanding over time toward oxygen-deficient environments (space and the stratosphere, underwater spaces and deep mines). Under such conditions, electric energy sources designed for various applications based on the chemical energy of hydrogen or other fuel oxidation by atmospheric oxygen inside electrochemical cells become impossible, so oxygen has to be replaced by another oxidizer as an energy supply. Such energy sources for mobile applications must possess a very high energy capacity for both transported reagents. With respect to reductant agent, hydrogen has become a top candidate, owing to the progress in development of its storage systems in compressed and bound forms (1.32 and 4.95 kW/dm^3^, respectively [1]). Thus, hydrogen should be combined with an oxidizer of a comparable energy density per unit mass/volume, in addition to the general requirements for operational safety.

Aqueous solutions of halogenates, i.e., salts of halogenic acids, wherein the halogen atom (X; X = Cl or Br) in the halogenate anion (XO_3_^−^) is of a high oxidation state (+5), represent evident candidates for such oxidants [2] in view of high theoretical energy content of aqueous solutions of such salts due to their good solubility and high halogenate/halide redox potential. However, their actual use inside power sources is inhibited by extremely low rates of halogenate electroreduction, even with specially modified electrodes.

It has been demonstrated in previous studies on bromate (MBrO_3_) salts, in acidic media [3,4,5,6] that this problem can be overcome, owing to the autocatalytic redox mediator mechanism of bromate reduction based on the bromine/bromide redox couple. This allowed us to construct efficiently operating hydrogen-bromate power sources for various operating modes [7,8]. Aqueous chlorate solutions can be considered even more suitable prospects than bromate solutions as multielectron oxidizers to construct electric power generators, in combination with the hydrogen oxidation reaction, owing to their higher theoretical energy density (Section 2), as well as their accessibility and cost-effectiveness.

Our initial strategy for the application of chlorate as an oxidant in power sources was to employ the same reactional scheme that had been successful for bromate, i.e., the autocatalytic mechanism based on a Cl_2_/Cl^−^ redox couple, which includes the reduction of Cl_2_ to Cl^−^ at the electrode surface and the comproportionation of ClO_3_^−^ and Cl^−^ in solution. However, the rates of both reactions are much lower than those for Br^−^-containing species. Moreover, the reaction between ClO_3_^−^ and Cl^−^ provides Cl_2_ as its single product only if excess amounts of Cl^−^ are present compared to ClO_3_^−^, whereas the relation between their concentrations inside the discharge device must be opposite, as Cl^−^ should play a catalytic role in the process.

Our recent analysis of chlorate electroreduction in aqueous acidic media [9] confirmed this argumentation, revealing that the ClO_3_^−^-to-Cl^−^ transformation proceeds via a different mechanism, whereby an important role is played by chlorine dioxide (ClO_2_). The principal objective of this study was to establish whether the rate of this process is sufficiently high to achieve specific power of the discharge, which is of interest for practical applications, as well as the maximum level of efficiency of transformation of the chemical energy into electricity.

## 2. Theoretical Estimation of Specific Energy of a Hydrogen-Chlorate Flow Generator: Potential Mechanisms of Chlorate Reduction

The cathodic process of hydrogen-chlorate battery consists of reduction of the halogenate anion (XO_3_^−^) to the corresponding halide ion (X^−^) due to six-electron transfer, with consumption of six hydrogen ions:

XO_3_^−^ + 6e^−^ + 6H^+^ = X^−^ + 3H_2_O (1)

This process is combined with electro-oxidation of molecular hydrogen at the gas-diffusional anode:

H_2_ − 2e^−^ = 2H^+^ (2)

Their combination inside the membrane-electrode assembly (MEA) might generate electric current in an external circuit, owing to the energy of the global chemical reaction:

XO_3_^−^ + 3H_2_ = X^−^ + 3H_2_O (3)

Electrode reactions are accompanied by hydrogen ion transfer across the separating cation-exchange membrane from anode to catholyte.

The energy source of such a current generator consists of a cylinder with compressed hydrogen and a reservoir with an aqueous solution of alkali metal halogenate. Circulation of the latter through the cathode chamber of the MEA leads to gradual depletion of the catholyte capacity due to a progressive increase in the halide/halogenate molar ratio from 0 to 1 due to reaction (1). The formation of water molecules in this reaction leads to a slight dilution of catholyte in its reduced state compared to its initial state.

The theoretical amount of specific energy contained per liter (or per kg) of the system, i.e., stored energy density (W_d_) is determined by the product of two quantities: specific redox charge of the system (C) and the potential difference between MEA electrodes (U^0^) at equilibrium:

W_d_ = U^0^ C (4)

The potential of each electrode deviates from the standard potential of its reaction, (1) or (2), due to deviations in the activities of their reagents and products from the standard values. Major correction of each standard potential originates from the non-unit activity of hydrogen ions in the corresponding medium in contact with the electrode. However, at equilibrium, the proton activities in catholyte and anolyte are identical, so the corresponding corrections of the potential difference (U^0^) are cancelled, i.e., it can be estimated as the difference between the standard potentials of reactions (1) and (2).

The specific redox-charge capacity of the system (C) in Equation (4) is obtained by summation of the inverse contributions of both electrodes, i.e., the specific redox-charge capacities of cathode (C^+^) and anode (C^−^):

C = C^+^ C^−^/(C^+^ + C^−^) (5)

These contributions (C^+^ and C^−^) are determined by the charge passing through an external circuit in the course of the corresponding electrode half-reaction (Equations (1) or (2)), which transforms the amount of the reagent inside 1 L of catholyte or anolyte in its reservoir, which is proportional to the saturated concentration of chlorate salt (C^+^ = 6 F c_sat_(ClO_3_^−^)) or to the maximal hydrogen density (ρ_max_(H_2_)) in the cylinder: C^−^ = 2 F ρ_max_(H_2_)/M(H_2_), where M(H_2_) is the hydrogen molar mass.

At room temperature, the density of the saturated LiClO_3_ solution is 1.97 g/cm^3^, and the solubility is 89.0%, i.e., c_sat_(ClO_3_^−^) = 19.3 M or 9.8 mol/kg solution [10]. The solubility of the potential reduction product (LiCl) is even higher. Under the condition of transferring six electrons to the LiClO_3_ particle during the cathodic process, the theoretical redox capacity is very high: C^+^ = 1580 Ah/kg of solution or 3100 Ah/dm^3^.

Solubilities of NaClO_3_ and Ca(ClO_3_)_2_ at room temperature are somewhat lower but still high: 4.7 and 6.3 mol of ClO_3_/kg of solution, respectively [11,12], which gives approximately: C^+^ = 760 and 1000 Ah/kg of solution.

All these indicators are much higher than those for the corresponding bromate salts; for example, the redox capacity of the saturated LiBrO_3_ solution is less than half of that for the LiClO_3_ solution.

Similarly to our previous studies of the bromate reduction process [2,3,4,7,8], a hydrogen anode was used in this study. At a pressure of 700 atm, the density of gaseous hydrogen at room temperature is ρ_max_(H_2_) = 40 g/dm^3^, which provides (even if the volume occupied by material of the gas cylinder is not taken into account) a charge density during its oxidation (C^−^) of only 1070 Ah/dm^3^. In comparison with the charge density of LiClO_3_ solution, the latter is about three times higher (at lower H_2_ pressures, this ratio is increased further).

Therefore, the total charge density of the hydrogen-chlorate battery (C) is relatively close to the charge density of hydrogen in the gas cylinder: about 800 Ah/dm^3^. If the standard potential of the chlorate-chloride transition (about 1.45 V vs. SHE) is used to estimate the standard cell voltage (U^0^), the stored energy density (W_d_) is about 1150 Wh/dm^3^, which is also essentially limited by the hydrogen reaction, even for the most advanced gas cylinders.

The same conclusion may be drawn if comparing the charge densities and energy capacities of a solution of LiClO_3_ and H_2_ in a gas cylinder per 1 kg of system.

The above consideration provides a merely ***thermodynamic*** analysis; it generates ***upper estimates*** for the redox-charge and energy densities of the hydrogen-chlorate discharge device within the framework of the ***assumption*** that it is possible to establish the conditions to perform chlorate reduction in conformity with Equation (1), i.e., ***up to the chloride ion***, and this process has a ***sufficiently high rate***. A priori, the validity of this assumption is far from being obvious, as the chlorate anion is ***non-electroactive*** within the required potential range (to ensure a decent voltage of power source), even on specially modified electrode surfaces, let alone inexpensive, non-modified carbon materials.

This problem is similar to that for hydrogen-bromate power sources, for which it was proposed [2] to search for a ***mediator redox cycle*** based on a redox couple (Ox/Red), whereby the Ox component is easily reduced at electrode to the Red form, whereas the latter is able to react with the principal oxidizer of the system to reduce it inside the solution phase (EC’ mechanism [13,14,15,16,17,18,19,20,21,22,23,24,25,26,27,28,29,30]). In the case of bromate reduction, the Br_2_/Br^−^ redox couple was indicated [2] as a prospective candidate:

Br_2_ + 2e^−^ = 2Br^−^        at electrode (6)

BrO_3_^−^ + 5Br^−^ + 6H^+^ = 3Br_2_ + 3H_2_O    in catholyte (7)

Theoretical analysis of coupled transport equations for the concentration distributions of all reactive components of the system (BrO_3_^−^, Br^−^, Br_2_ and H^+^) (performed for various transport models: Nernst stagnant layer [31], generalized Nernst layer [32], convective–diffusional transfer [33] and convective–migration–diffusion transport [34]) revealed that the process described by Equation (6) and (7) does ***not*** belong to the EC’ mechanism, although this reactional scheme is based on a redox-mediator cycle, as it represents an example of an ***autocatalytic*** process whereby the principal reactant (BrO_3_^−^) is transformed ***not*** into an ***inert product*** but into ***components of the mediator redox couple*** (Br_2_/Br^−^). As a result, under suitable transport conditions, the current passage leads to accumulation of high concentrations of these catalytic species (Br_2_ and Br^−^) near the electrode surface, accompanied by a strong current that it is limited by the transport of the principal components of the bulk solution (BrO_3_^−^ and H^+^), despite their non-electroactivity on the electrode surface.

These theoretical predictions were later confirmed by experimental studies of this process at the rotating disk electrode [4] and microelectrodes [35]. The possibility of strong currents in solutions containing very low bulk-solution concentrations of the catalytic species (Br_2_) was also supported by a study of hydrogen-bromate MEA electricity sources, which revealed very high current and power densities, as well as almost complete transformation of bromate into bromide, within a single passage of solution through the discharge device [7,8].

In view of the parallelism of the properties of Br^−^- and Cl^−^-containing compounds, the same autocatalytic reaction mechanism (Equation (6) and (7)) could be considered for the chlorate reduction process:

Cl_2_ + 2e^−^ = 2Cl^−^   at electrode (8)

ClO_3_^−^ + 5Cl^−^ + 6H^+^ = 3Cl_2_ + 3H_2_O  in catholyte (9)

However, experimental studies of this comproportionation reaction (Equation (9)) [36,37,38,39,40,41,42,43] show that it is much slower than that for bromate (Equation (7)). This reaction route (Equation (9)) is also only predominant for high Cl^−^ concentrations, whereas the same reactants follow another route for low chloride content [36,37,38,39,43]:

ClO_3_^−^ + Cl^−^ + 2H^+^ = ½Cl_2_ + ClO_2_ + H_2_O in catholyte (10)

In particular, such a situation takes place in the case of chlorate electroreduction, at least for the initial period of accumulation of various reduction products where the chloride concentration is very low compared to the chlorate concentration.

If reaction (10) takes place inside the electrochemical cell, then not only Cl_2_ but also ClO_2_ might be reduced on the electrode surface:

ClO_2_ + H^+^ + e^−^ = HClO_2_  at electrode (11)

whereas HClO_2_ is subject to further disproportionation steps that are strongly dependent on the concentrations of Cl compounds of various oxidation degrees [9].

This means that the expected mechanism of chlorate reduction is much more complicated than the combination of Equations (8) and (9), so that it is not evident a priori whether its rate is sufficiently high for application in electric power sources.

To avoid additional complications related to saturated solutions of high concentrations, in this study, we considered ***moderate*** concentrations of chlorate (from 1 M to 3 M NaClO_3_) in this study. Preliminary tests showed that the reaction rate is too slow for concentrations of acid similar to those for H_2_-BrO_3_^−^ batteries”. To accelerate the reaction, experiments were carried out for higher acidities: from 4 M to 6 M H_2_SO_4_.

## 3. Results and Discussion

### 3.1. Measurement of Voltammetric Characteristics of Discharge Cells of Hydrogen-Chlorate MEA for Various Electrolyte Solution Compositions

**Figure 1:**
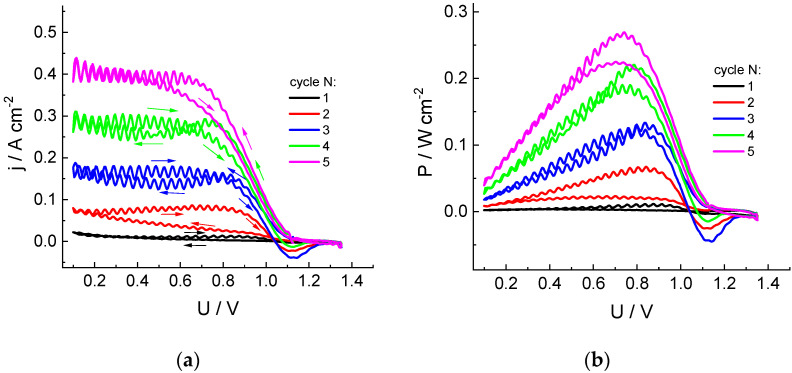
Dependences of the cathodic current density (**a**) and specific power (**b**) of the discharge on cell voltage for five successive cycles of voltage sweep from OCV to 0.1 V for the hydrogen-chlorate MEA: Nafion 212 membrane (working area: 4 cm^2^); cathode: three sheets of carbon paper (SIGRACET 39AA): anode: GDL Freudenberg H24C5C13, Pt 0.47 mg/cm^2^: catholyte composition: 1 M NaClO_3_ + 4 M H_2_SO_4_; circulation rates: 54 mL/min (solution), 2.5 dm^3^/h (H_2_); voltage sweep rate: 5 mV/s. The first scan starts from the fresh solution in the reservoir.

Figure 1 shows dependences of current (j) and power density (P) on polarizing voltage (U) for a cell with an MEA area of 4 cm^2^ in the course of five successive voltage sweep cycles in the range of 1.4 to 0.1 V and back for chlorate-sulfuric acid catholyte circulating through a porous cathode at a rate of 54 mL/min. During the first pass from OCV (around 1.1 V) to 0.1 V (reservoir contains freshly prepared electrolyte), almost zero current is observed, with a slight increase near the lower sweep limit.

In the course of the first backward voltage sweep, as well of the subsequent scans, there is a gradual increase in the current and discharge power compared to the previous scan. Low-amplitude pulsations visible in these curves are due to pressure surges of the pump supplying the electrolyte into the electrode space.

This progressive increase in the cathodic current amplitude evidently originates from the change in the solution composition inside reservoir, with accumulation of catalytically active species, which accelerate the chlorate reduction process.

Special attention should be paid to the appearance of currents of the ***opposite polarity***, which are observed at sufficiently large positive voltages ***during the backward scans***, whereas they are ***absent*** within the cathodic scans. This observation implies the formation of chlorate reduction products of lower oxidation degrees and their accumulation near the electrode surface, which are able to be reoxidized at sufficiently positive voltages. Changes in the current polarity take place in the voltage range of 1.0 to 1.1 V, whereas the maximal anodic current is observed in the range of 1.1 to 1.15 V. These data imply that the anodic current is related to the process following: HClO_2_ − e^−^ = ClO_2_ + H^+^, i.e., oxidation of chlorous acid, as the standard potential of this process is 1.14–0.059 pH [10].

The intensity of this effect increases during the first three cycles; then, the opposite tendency is observed: diminution of the amplitude of the anodic current compared to the previous cycle, contrary to the continuing increase in the cathodic current at lower voltages. This behavior might indicate the appearance of additional chemical reactions inside the electrolyte solution or transition of the mediator role to another redox pair.

**Figure 2:**
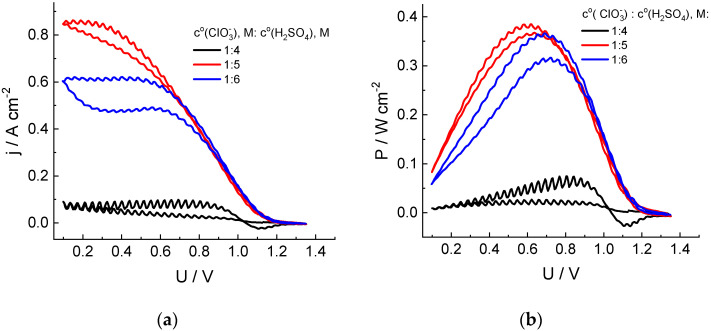
Dependences of the cathodic current density (**a**) and specific power (**b**) of the cell discharge on its voltage in the course of the second cycle of its sweep from OCV to 0.1 V and back for hydrogen-chlorate MEA. Catholyte composition: 1 M NaClO_3_ + x M H_2_SO_4_ (x = 4, 5 or 6). See Figure 1 for additional information.

The increase in the acid content in the electrolyte (Figure 2) leads to a significant increase in the discharge currents. For the chlorate electrolyte in 5 M acid, the discharge power reaches 1.5 W (surface area: 4 cm^2^) by the second cycle of the cell voltage sweep, compared to 0.25 W for 4 M H_2_SO_4_. On the contrary, a further increase in the acid content to 6 M leads to a decrease in the discharge currents and power compared to those for 5 M acid.

The extremal character of the dependence of the chlorate anion reduction rate in the presence of chlorine dioxide as an intermediate on the acid concentration was also reported in Refs [44,45], with the maximal rate within the range of 4.5–5.5 M. It was attributed to superposition of the acidity effects on processes of dis- and comproportionation between chlorine compounds of various oxidation degrees. Furthermore, the electrical conductivity of the sulfuric acid solution passes through its maximum within this concentration range [46].

The following sections are devoted to questions on how deeply chlorate can be converted with such power and which chlorine compounds are involved in this increase in electrical activity.

### 3.2. Measurement of Discharge Characteristics of Hydrogen-Chlorate MEA Cells for Various Electrolyte Solution Compositions: Complete Chlorate Transformation at a Voltage Equal to 0.7 V

**Figure 3:**
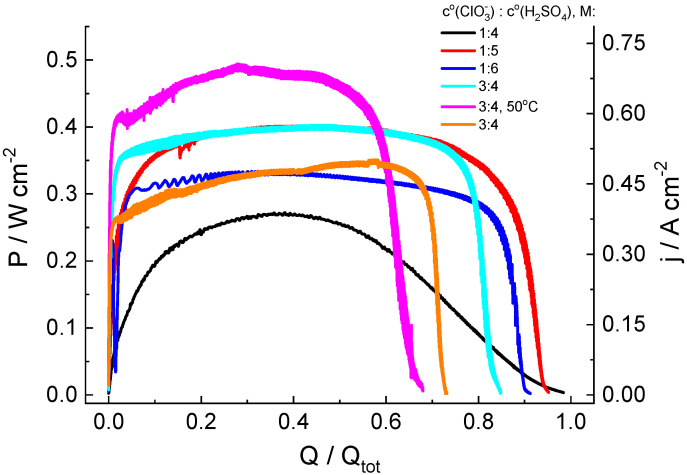
Discharge curves for hydrogen-chlorate MEA cells under a potentiostatic load of 0.7 V for catholyte solutions of various compositions: y M NaClO_3_ + x M H_2_SO_4_ (y = 1 or 3; x = 4, 5 or 6; indicated in legend for each graph). See Figure 1 for additional information.

Figure 3 shows the evolution of the electric power generated by the cell in the course of the complete electrolytic conversion of the catholyte solution for its various compositions in long-term potentiostatic experiments. The x-axis in Figure 3 is the ratio of the charge (Q) passed through the cell to its total value (Q_tot_) calculated for the complete conversion of all redox-active electrolyte compounds into the reduced form, i.e., chlorate ClO_3_^−^ to chloride Cl^−^.

In the chlorate-acid system, reduction of the non-electroactive chlorate anion takes place via chlorine compounds of intermediate oxidation states [44,45], which are accumulated in solution, owing to chlorate reduction, which makes it possible to assume a priori an autocatalytic character of the process, resulting in a significant increase in the generated current under potentiostatic load, which is replaced by current decrease in a subsequent stage due to the depletion of chlorate. This expectation was confirmed in the course of our measurements. However, both the rate of current increase and its maximal value significantly depend on the initial composition of catholyte.

Potentiostatic load plots for catholytes of various compositions (Figure 3) measured under identical experimental conditions with discharge cells of the same construction were used to compare rates of the current-generating reaction and its efficiencies for these solutions by calculation of several parameters for each plot:

- **Faradaic efficiency, FE**: ratio of the charge (Q_max_) passed during the experiment to its calculated total value based on the initial chlorate concentration, c^0^(ClO_3_^−^): Q_tot_ = 6Fc^0^(ClO_3_^−^)V_sol_, where V_sol_ is the catholyte volume: FE = Q_max_/Q_tot_;

- **Energy efficiency, EE**: ratio of the total generated electric energy to the calculated chemical energy expressed by the following formula: W_tot_ = U^0^Q_tot_, where the standard cell voltage (U^0^ = 1.449 V) is equal to the standard electrode potential for the reaction: ClO_3_^−^ + 6H^+^ + 6e^−^ ⇄ Cl^−^ + 3H_2_O [47] vs. SHE;

- **Maximal specific power of discharge, P_max_**: maximal specific power of the cell;

- **Average specific power of discharge, P^90^_av_**: average specific power generated within the period before 90% depletion of the catholyte capacity;

- Time for the discharge power to reach the average value, t(P = P^90^_av_);

- **Residual power, P_rest_**: specific discharge power at the moment when 90% of the total charge (0.9 Qtot) has passed through the cell; and

- Time of 90% depletion of catholyte capacity, t_rest_.

Values of these parameters derived from the data presented in Figure 3 are given in Table 1.

According to data therein, the following conclusions can be drawn:

1. For the 1 M NaClO_3_ + 5 M acid solution, high values of the maximal specific discharge power (P_max_ = 402 mW/cm^2^ at room temperature) and of the average discharge power during the time interval up to the use of 90% of the catholyte capacity (P^90^_av_ = 348 mW/cm^2^) were recorded. With respect to energetic efficiency, its value (EE = 0.46) mostly reflects the voltage (0.7 V) at which the electricity was generated, compared to the standard potential of the ClO_3_^−^-to-Cl^−^ reaction (E^0^ = 1.449 V), i.e., it might be strongly enhanced at a higher functioning voltage.

2. Dependence of the power characteristics of the cell on the acid concentration were non-monotonous, i.e., their values for 4 M and 6 M acid solutions were much lower than those for the 5 M acid solution.

3. The faradaic efficiency (FE) for all studied systems with 1 M chlorate content exceeded 90%. An increase in chlorate concentration in the catholyte leads to lower FE values. A possible reason for the decrease in FE for 3 M NaClO_3_ solutions (compared to that for 1 M solutions) is a proportional increase in the amount of intermediate (ClO_2_) in the reservoir, both in catholyte solution and inside the gas phase above it. The latter increases the rate of chlorine dioxide absorption processes by plastic components of the electrolyte storage and supply system, as indicated by an intensive yellow color characteristic of chlorine dioxide on surfaces of the peristaltic pump pipe, the polypropylene tank cover and of the PTFE connections at the end of experiments. The increase in ClO_3_^−^ concentration can also be accelerated by the disproportionation rate of its reduction product (HClO_2_) owing to its second-order kinetics, thus reducing the concentration of the mediator redox couple (ClO_2_/HClO_2_).

4. The temperature increase in the electrolyte solution and of the discharge cell to 50 °C enables significant growth of the discharge power (from 400 to 493 mW/cm^2^) and some reduction in time to reach the average level (from 18 to 16 s). However, these advantageous effects of higher temperature are counterbalanced by a decrease in the faradaic efficiency (FE) by 17%.

To verify whether the drop in faradaic efficiency (FE) is a consequence of the crossover of chlorine compounds through the membrane into the anodic compartment, the hydrogen leaving the anode space was passed through a hydraulic seal containing 40 mL of distilled water to trap the reduced chlorine compounds in the form of HCl (see Section 4 for details). Its content after completion of each potentiostatic load experiment was determined with square wave voltammetry on a platinum electrode to register the chloride oxidation peak. Results of this analysis for catholytes of various compositions are summarized in Table 2.

A comparison of columns 3 and 4 of Table 2 shows that the transmembrane crossover of chlorine compounds to the anode compartment in the course of load experiments is responsible for only a small fraction of faradaic losses (2.5 to 4.5%) for various catholytes. The dominant fraction of the unconverted electrolyte capacity can be attributed to a significant retardation of the chlorate reduction due to a decrease in its concentration at the end of the process, as well as to the formation of solute chlorine compounds that do not react on the cathode at a cell voltage of 0.7 V.

To determine how the crossover of chlorine-containing compounds in the course of load measurements affected the state of the platinum catalyst of anode, we performed a voltametric study of the electrochemical sorption/desorption of hydrogen in order to measure the electrochemically active surface area (ECSA) of dispersed platinum. A specific feature of this measurement inside the hydrogen-chlorate system is the necessity to fill in the cathodic space with a solution containing components of a reversible redox couple in order to fix the cathode/catholyte interfacial potential (due to the absence of a catalytic layer on the cathode, the hydrogen oxidation-reduction reaction cannot be used for this purpose as is done inside hydrogen-air fuel cells).

**Figure 4:**
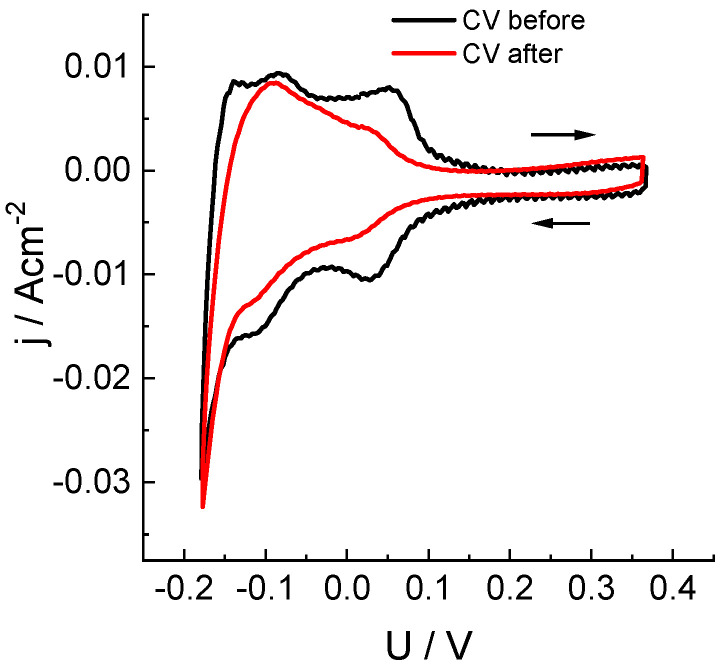
Study of electrochemical adsorption/desorption of hydrogen on a Pt/C anode catalyst (see Figure 1 for information) before and after five potentiostatic load tests at 0.7 V (see Figure 3 and Table 1) by means of cyclic voltammetry: voltage sweep rate: 50 mV/s, sweep limits: −0.18 to 0.37 V. Voltage was measured between the cathode in contact with the half-reduced solution of 0.15 M 2,7-Na_2_AQDS (1 M H_2_SO_4_) and the anode with a Pt/C catalyst in an argon atmosphere.

In our experiments, anthraquinone-2,7-disulfonic acid sodium salt (Na_2_AQDS) was used as a redox couple. Measurements of hydrogen adsorption/desorption on the MEA platinum catalyst were performed on an anode purged with argon. Its potential was swept with respect to the practically non-polarizable (under measurement conditions) positive electrode in contact with an Na_2_AQDS solution. The obtained results (Figure 4) characterize the change in ECSA of the anode catalyst before and after five load tests performed on the MEA cell without replacement of the catalytic layer. The observed effect of diminution of the ECSA area (by 33%) can be attributed to both a partial dissolution of platinum particles under aggressive action of chlorine compounds that were transferred across the membrane and to the partial closure of active centers.

### 3.3. UV-Visible Spectroscopy Analysis of Electrolyte Solution Composition at the Exit of the Cathode Compartment of a Hydrogen-Chlorate MEA Battery in the Course of its Functioning under Various Regimes

**Figure 5:**
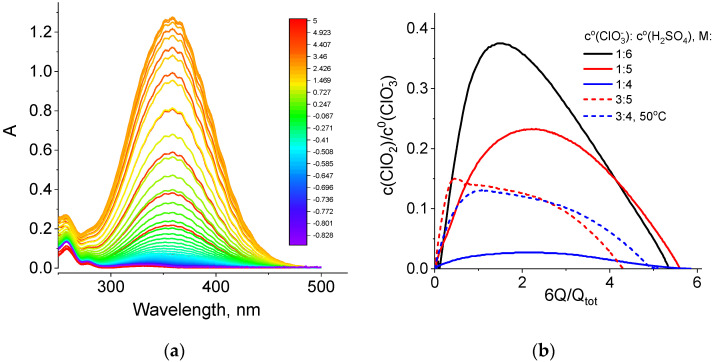
(**a**) Absorption spectra of the NaClO_3_ + 4 M H_2_SO_4_ catholyte (optical path length: 380 μm) in the course of the potentiostatic load experiment at a voltage of 0.7 V (black curve in Figure 3, line 1 of Table 1). Scale colors in the figure indicating colors of spectral lines correspond to the average oxidation degrees of Cl atoms in solution, which was calculated by the following formula: 5 − Q/Q_tot_. (**b**) Dependence of the relative content of chlorine dioxide inside the catholyte (c(ClO_2_)/c^0^(ClO_3_^−^), where c^0^(ClO_3_^−^) is the initial concentration of chlorate) on the value of the transferred charge (Q) expressed in redox equivalents of chlorate anions, (6Q/Q_tot_).

During all potentiostatic load experiments (Figure 3 and Table 1), absorption spectra of the catholyte in the optical and UV wavelength ranges were recorded every 10 s with a flow cuvette inserted into the positive electrode line (Figure S2). Such spectra for one of the studied catholyte compositions are shown in Figure 5a.

We found that the strongest change in absorption intensity during the chlorate cathode operation is related to variation in the amplitude of the chlorine dioxide band, with a maximum at 358 nm. A band with a maximum at 260 nm and a characteristic shoulder on the side of higher wavelengths was assigned to sodium chlorite, i.e., salt of chlorous acid (HClO_2_) in Ref. [48]. From the series of spectra of this type for each discharge experiment at 0.7 V, data on the dependence of the chlorine dioxide concentration on the amount of passed charge equivalents were extracted for catholytes of various chlorate and acid concentrations (Figure 5b).

For all five experiments with chlorate electrolytes, these dependences have a qualitatively similar shape: initially, an approximately linear growth, then passage through a maximum and, finally, a decrease until the catholyte capacity is almost completely exhausted, while the ClO_2_ concentration approaches zero. Table 3 shows slopes of the initial segments of the dependences in Figure 5b recalculated in terms of the number of chlorine dioxide molecules that are added to the chlorate-acid catholyte if one electron is passed through the circuit. These values are less than one (0.03 to 0.5 molecules per electron) and vary considerably depending on the composition of the electrolyte.

This result supports the existence of several kinetic routes for formation of chlorine dioxide from chlorate and the product of its electrochemical reduction, chlorous acid (HClO_2_), so the contributions of these routes depend on the composition of the medium. According to Ref [49], the set of chemical steps required for branching the process of chlorine dioxide formation should include at least five reactions between various oxygen-containing compounds:

ClO_2_ + H^+^ = HClO_2_ + e, (12)

HClO_2_ + HClO_3_ = 2ClO_2_ + H_2_O, (13)

2HClO_2_ = HClO_3_ + HClO, (14)

HOCl + HClO_2_ = HClO_3_ + HCl, (15)

HOCl + 2HClO_2_ = 2ClO_2_ + HCl + H_2_O. (16)

If ClO_2_ is reduced in acidic medium in the presence of chlorate ions via step (12), the reaction generates an intermediate species with a very unstable oxidation degree (Cl(+3) HClO_2_), which can participate in numerous subsequent steps. The comproportionation reaction (13) between ClO_3_^−^ and HClO_2_ provides two ClO_2_ molecules so that reactions (12) and (13), together, represent an autocatalytic cycle, which could lead to the exponential accumulation of ClO_2_ in solution from chlorate [9]. In parallel, HClO_2_ molecules can participate in the disproportional step (14), producing another unstable and therefore very active catalytic HClO molecule, which can react with HClO_2_ (step 15), generating a Cl^−^ ion. The latter is known as an active catalyst, strongly accelerating step (16). As a result, kinetics of transformations in this system depend strongly on its initial composition, as well as other factors, such as temperature.

Another origin of experimental operando information with respect to the composition of the catholyte in the course of its redox transformation is represented by the potential of an indicator electrode in contact with solution under open-circuit conditions so that its potential is determined by the Nernstian equation for the principal redox couple; therefore, this parameter characterizes the redox potential of the solution.

**Figure 6:**
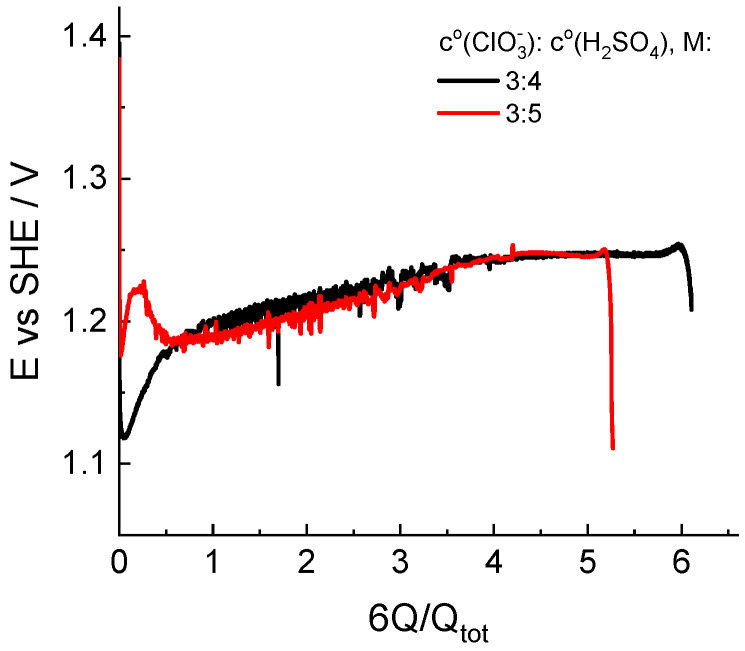
Dependence of the redox potential of the solution measured by an indicator electrode in the catholyte reservoir in the course of potentiostatic load experiments at a voltage of 0.7 V. Initial compositions of the catholyte are indicated in the legend.

Figure 6 illustrates the dependence of the indicator electrode potential on the depth of chlorate catholyte reduction for two initial compositions: 3 M NaClO_3_ + 4 M H_2_SO_4_ and 3 M NaClO_3_ + 5 M H_2_SO_4_. The behavior of the electropotential indicator is quite different in the initial stage of the process, even for solutions of relatively similar compositions.

Throughout both experiments, the indicator potential was established in the vicinity of the standard potential of the redox couple (ClO_2_/HClO_2_): 1.14–0.059 pH vs. SHE in V, which is far from that of the Cl_2_/Cl^−^ couple: 1.396 V [47]. In combination with results of the spectrophotometric analysis of the composition, this circumstance allows us to consider the route of conversion of chlorate to chloride via chlorine dioxide as the principal route.

## 4. Materials and Methods

The performed experiments included electrochemical measurements of current voltage and load characteristics of the cell containing a membrane-electrode assembly (MEA). Two electroactive media were supplied to the MEA electrodes: hydrogen (from a GVCh 36A hydrogen generator, NPP Khimelektronika, Moscow, Russia) and aqueous solution of sodium chlorate (NaClO_3_, 99+%, Acros Organics, Fair Lawn, NJ, USA) and sulfuric acid (H_2_SO_4_, chemically pure, Himmed, Moscow, Russia). The solution was prepared by dissolving a weighed portion of sodium chlorate and an aliquot of sulfuric acid in tridistilled water (UD-3015, ULAB, Moscow, Russia) until the required volume was obtained.

The MEA of the hydrogen-chlorate battery included two carbon electrodes separated by a Nafion 212 perfluorinated cation-exchange membrane (DuPont). The membrane was used without pretreatment. A negative electrode for oxidation of gaseous hydrogen was prepared by depositing a layer of Pt/C catalyst (50% metal content, Prometheus, Rostov-on-Don, Russia) on Freudenberg carbon paper. Catalyst loading was 0.5 mg per 1 cm^2^ of membrane in terms of platinum. The composition of the catalytic ink also included a Nafion ionomer solution, with an N:C ratio of 0.7. To ensure contact of the ionomer inside the layer with the membrane, carbon paper with a catalytic layer was pressed on the membrane (pressure of 80 kg/cm^2^, temperature: 80 degrees Celsius) for 3 min. Positive electrodes were collected from several sheets of SIGRACET 39AA carbon paper (SGL Carbon) folded into a stack and pressed mechanically from the opposite (with respect to the catalytic layer) side of the membrane in such a way that the compression degree of the positive electrode along the direction normal to the membrane surface was about 20%.

The described MEA was tested in a cell of homemade construction (Figure S1), which included end plates (titanium) with pressed fittings (PVDF) for hydrogen and chlorate catholyte supply. Current-collecting plates from the side of the negative electrode were made of three sheets of Graflex carbon foil (Unihimtek, Moscow, Russia) with channels for distribution of the hydrogen flow throughout the porous anode, which formed an “interdigitated”-type flow field [50]. The current collector plate for the positive electrode was made of glassy carbon and equipped with a large-area pressure metal contact. The catholyte was fed into the electrode space and distributed over the porous carbon cathode with the use of holes and a flow channel of “serpentine” geometry made of glassy carbon by laser engraving. Frames that restricted the flow were made of Teflon sheets (anode) or perfluorinated rubber Viton (cathode), with square slots to place MEA electrodes. Their thickness regulated the compression degree of the electrode materials.

To carry out measurements with a hydrogen-chlorate MEA under various regimes, with parallel analysis of the catholyte solution composition, an installation was assembled (Figure S2) that included a discharge cell (electrode area: 4 cm^2^) connected to chlorate solution reservoir by a line of Teflon tubes (internal diameter: 1.6 mm). Electrolyte solution was supplied to the positive electrode chamber of the cell by a peristaltic pump (BT100-1L, Longer Precision Pump Co., Ltd., Baoding, China). Circulation of the solution ensured passage of the entire chlorate electrolyte volume through the cell for 10–12 s, leading to a rapid averaging of the concentrations of all solute components (including reagents and products of electrodes and homogeneous reactions) through the entire volume of solution. A flow spectrophotometric cuvette of homemade construction of variable optical path (0.2–2 mm) was inserted into the line between the pump and the cell, recording the solution spectra inside the cuvette with a fiber optic spectrophotometer (Avaspec ULS2048CL-EVO-RS, Avantes B.V., Apeldoorn, The Netherlands). The solution leaving the cell was returned to the reservoir, closing the circulation loop. The anode of the cell was connected through an additional pressure reducer, owing to which a constant overpressure of hydrogen of 20–30 mbar was maintained at the cell inlet. Hydrogen was released from the cell into the atmosphere through a hydraulic lock with a water volume of 40 mL equipped with gas flow regulator, which ensured a supply of 30 mL/min. Owing to such a functioning procedure, more intensive consumption of hydrogen at the anode was automatically compensated by acceleration of its supply to the circuit so that the anode space was purged with gas to remove the crossover products of the catholyte components at a constant rate, regardless of consumption.

To determine the total amount of chlorine compounds that penetrated into the anode chamber in the course of long-term discharge experiments, the composition of water in the hydraulic lock was analyzed. Passing through the anode catalytic layer under a hydrogen atmosphere, these chlorine compounds were reduced to HCl, which was trapped in the water of the hydraulic lock. The chloride anion content was measured by pulse electroanalytical method after preliminary calibration of the dependence of the intensity of the oxidation peak on the chloride content (see Figure S3).

MEA polarization was performed with an Autolab 302N potentiostat (Metrohm AG Herisau, Switzerland) with a FRA2 module and a 20A booster. For each of the studied compositions of the chlorate electrolyte solution, the sequence of electrochemical measurements included:

1. Measurement of cell impedance under conditions of hydrogen supplied to the anode while freshly prepared catholyte solution passed through the cathode at open-circuit voltage (OCV) in order to determine the ohmic resistance (to verify the quality and reproducibility of the assemblage of the installation).

2. Measurement of the current-voltage characteristic of the cell in the regime of cyclic voltage sweep from OCV to 0.1 V under the same conditions of hydrogen and chlorate solution supply to the electrode in order to determine the voltage corresponding to the maximal discharge power.

3. Measurement of the load curve at the constant voltage determined in the previous stage, up to complete exhaustion of the redox capacity of a fresh portion of the electrolyte solution, with parallel registration of absorption spectra of the solution in the course of its transformation. To measure the dependence of the redox potential of the solution on the depth of electrolysis, two additional electrodes were introduced into the reservoir: an indicator (platinum grid) and a reference electrode (silver chloride); the potential difference between these two electrodes was recorded by a high-resistance voltmeter synchronized in time with a potentiostat to polarize the cell.

In some experiments, additional stages of monitoring of the state of the platinum catalyst inside the MEA were performed before and after measurements 1 to 3. An original procedure was proposed and elaborated for these stages. Acidified solution of compounds possessing a very high electron transfer rate, anthraquinone 2,7-disulfonic acid (AQDS, >97%, TCI), which contains equivalent amounts of its quinone and hydroquinone forms, was fed to the cathode instead of chlorate solution. Owing to electrochemical equilibrium of the quinone and hydroquinone forms at the cathode, its electrode potential value was fixed at about 0.21 V vs. SHE. The anode compartment, including the Pt/C catalytic layer, was filled with inert gas (Argon, 99.999, Linde Gas Rus, Moscow, Russia). The cell was connected to a potentiostat, and the anode was polarized in the CV regime within the potential range corresponding to the formation and electrochemical desorption of hydrogen on the platinum surface, assuming the cathode potential to be constant.

## 5. Conclusions

The principal result of this study is the demonstration, for the first time, of the construction of a hydrogen-chlorate electricity generator that possesses a sufficiently high rate in terms of the discharge current density (up to 0.7 A/cm^2^), specific discharge power (over 0.4 W/cm^2^) and current efficiency of the chlorate-to-chloride transformation (up to 99%). The above values of discharge currents and specific power of the H_2_–ClO_3_^−^ device are a factor of three to four lower than those for H_2_–air PEMFC with a proton-exchange membrane of the Nafion type (the most constructively similar FC type). According to a review by Ren et al. [51], specific power of the latter is about 1.5 W/cm^2^ for platinum metals on carbon substrates as catalysts, whereas it exceeds 2 W/cm^2^ if air is replaced by pure oxygen. Keeping in mind the difference in “the level of maturity” of the devices under consideration, the difference in parameters of these devices is not large, so the chlorate cathode can be considered sufficiently “rapid” for its use in combination with molecular hydrogen oxidation at the anode inside for mobile applications in airless environments.

Operando spectrophotometric analysis of the catholyte composition inside the discharge cell showed that its functioning is based on the autocatalytic redox mediator mechanism, whereby chlorine dioxide is accumulated in solution and plays a key role in the chlorate reduction process. In contrast to the previously described bromate reduction in acidic solution, which proceeds via a bromide/bromine mediator couple, the chloride/chlorine redox couple does not make a comparable contribution to the overall rate of the process in acidized chlorate electrolyte.

We found that other oxo compounds of chlorine are also involved in the autocatalytic cycle of chlorate reduction inside the hydrogen-chlorate MEA cell; in particular, spectrophotometry provides evidence in favor of the presence of chlorous acid inside the catholyte. The combination of spectrophotometric and coulometric data with the results for the redox potential of the solution (potentiometry of a platinum indicator electrode) revealed a strong dependence of the chlorate electroreduction route on the composition of the solution, which is a consequence of several parallel dis- and comproportionation routes involving chlorine dioxide, chlorous acid and hypochloric acid.

It has been established that despite the low level of crossover of chlorine compounds through the membrane (capacitance losses associated with this phenomenon are less than 5% of the total faradaic losses), there is a noticeable decrease in the electrochemically active surface area of the platinum catalyst after long-term load tests. Determination of the service life of the components of the hydrogen-chlorate MEA cell, including the catalyst for the hydrogen oxidation reaction at the anode, will be a subject of our future studies.

## Figures and Tables

**Table 1:** Discharge characteristics of cells (chlorate cathode and hydrogen gas diffusion anode) recorded at a constant voltage of 0.7 V. Membrane-electrode unit surface area: 4 cm^2^; catholyte volume: 10 mL; catholyte circulation rate: 54 mL/min.

N	c^0^(ClO_3_^−^)	c^0^(H_2_SO_4_)	FE	EE	P_max_	P^90^_av_	t(P > P^90^_av_)	P_rest_	t_rest_
	M	M	-	-	mW/cm^2^	mW/cm^2^	min	mW/cm^2^	min
1	1	4	0.99	0.48	289	145	16	31	103
2	1	5	0.95	0.46	402	348	7	308	41
3	1	6	0.91	0.44	334	293	6	275	47
4	3	4	0.85	0.41	400	376	18	351	123
5 ^1^	3	4	0.68	0.33	494	447	16	255	83
6	3	5	0.73	0.35	351	315	44	327	127

^1^ The experiment was carried out at an elevated temperature of the catholyte and the MEA cell (50 degrees Celsius).

**Table 2:** Amount of chloride anion inside the hydraulic seal (the technique is described in SM) at the outlet of the anode space of the hydrogen-chlorate cell after the end of each potentiostatic loading experiment at 0.7 V and comparison with the value of the faradaic loss of charge (1-FE) (Q_tot_) for the same experiment.

Initial Catholyte Composition		Concentration of Cl Atoms inside the Hydraulic Seal (40 mL) after H_2_ Anode, mM	Total redox Charge of Cl^−^-Containing Components ^1^ (for Their Transformation to Cl^−^) Transferred from Catholyte (C)	Faradaic Losses, (1-FE) (Q_tot_) Based on Data in the FE Column of Table 1,C
c^0^(ClO_3_^−^), M	c^0^(H_2_SO_4_), M			
1	5	0.65	12.5	288
1	6	1.22	23.5	518
3 ^2^	4	2.40	46.3	1843
3	5	3.36	64.8	1555

^1^ Calculated on the basis of the assumption that the main contribution to the flow of chlorine-containing particles through the membrane is provided by chlorine dioxide. ^2^ The experiment was carried out at an elevated temperature of the catholyte and the MEA cell (50 degrees Celsius).

**Table 3:** Change in the number of ClO_2_ molecules inside the catholyte per electron passed through the cell in the initial stage of the potentiostatic load test of the MEA cell for catholytes of various chlorate/acid molar ratios.

c^0^(ClO_3_^−^), M	c^0^(H_2_SO_4_), M	dc(ClO_2_)/dn_e_, Initial Stage
1	4	0.03
1	5	0.2
1	6	0.5
3 ^1^	3:4	0.26
3	3:5	0.5

^1^ The experiment was carried out with an elevated temperature of the catholyte and the MEA cell (50 degrees Celsius).

## Supplementary Materials

The following supporting information can be downloaded at: https://www.mdpi.com/article/10.3390/molecules27175638/s1, Figure S1: Design of the discharge cell (surface area: 2 × 2 cm^2^); Figure S2: Scheme of the experimental setup (cathode circuit); Figure S3: Square-wave voltammogram (a) and calibration curve of coordinates: peak current vs. chloride anion concentration (b).

## References

[1] Hassan I.A., Ramadan H.S., Saleh M.A., Hissel D. (2021). Hydrogen storage technologies for stationary and mobile applications: Review, analysis and perspectives. Renew. Sustain. Energy Rev..

[2] Tolmachev Y.V., Piatkivskyi A., Ryzhov V.V., Konev D.V., Vorotyntsev M.A. (2015). Energy cycle based on a high specific energy aqueous flow battery and its potential use for fully electric vehicles and for direct solar-to-chemical energy conversion. J. Solid State Electrochem..

[3] Vorotyntsev M.A., Antipov A.E., Konev D.V. (2017). Bromate anion reduction: Novel autocatalytic (EC″) mechanism of electrochemical processes. Its implication for redox flow batteries of high energy and power densities. Pure Appl. Chem..

[4] Modestov A.D., Konev D.V., Antipov A.E., Petrov M.M., Pichugov R.D., Vorotyntsev M.A. (2018). Bromate electroreduction from sulfuric acid solution at rotating disk electrode: Experimental study. Electrochim. Acta.

[5] Cho K.T., Razaulla T. (2019). Redox-mediated bromate based electrochemical energy system. J. Electrochem. Soc..

[6] Chinannai M.F., Ju H. (2021). Analysis of performance improvement of hydrogen/bromine flow batteries by using bromate electrolyte. Int. J. Hydrogen Energy.

[7] Modestov A.D., Konev D.V., Tripachev O.V., Antipov A.E., Tolmachev Y.V., Vorotyntsev M.A. (2018). A Hydrogen–Bromate Flow Battery for Air-Deficient Environments. Energy Technol..

[8] Modestov A.D., Konev D.V., Antipov A.E., Vorotyntsev M.A. (2019). A Hydrogen-bromate flow battery: Can one reach both high bromate utilization and specific power?. J. Solid State Electrochem..

[9] Konev D.V., Goncharova O.G., Tolmachev Y.V., Vorotyntsev M.A. (2022). Role of chlorine dioxide in the process of clorate electroreduction at low pH. Russ. J. Electrochem..

[10] Campbell A.N., Paterson W.G. (1958). The conductances of aqueous solutions of lithium chlorate at 25.00 °C and at 131.8 °C. Can. J. Chem..

[11] Campbell A.N., Kartzmark E.M., Maryk W.B. (1966). The sustems sodium chlorate-water-dioxane and lithium chlorate-water-dioxane, at 25 °C. Can. J. Chem..

[12] Hanley J., Chevrier V.F., Berget D.J., Adams R.D. (2012). Chlorate salts and solutions on Mars. Geophysi. Res. Lett..

[13] Fleischmann M., Lasserre F., Robinson J., Swan D. (1984). The application of microelectrodes to the study of homogeneous processes coupled to electrode reactions: Part I. EC0 and CE reactions. J. Electroanal. Chem. Interfacial Electrochem..

[14] Compton R.G., Day M.J., Laing M.E., Northing R.J., Penman J.I., Waller A.M. (1988). Rotating-disc electrode voltammetry. The catalytic mechanism (EC’) and its nuances. J. Chem. Soc. Faraday Trans. 1 Phys. Chem. Condens. Phases.

[15] Denuault G., Fleischmann M., Pletcher D., Tutty O.R. (1990). Development of the theory for the interpretation of steady state limiting currents at a microelectrode: EC’ processes: First and second order reactions. J. Electroanal. Chem. Interfacial Electrochem..

[16] Denuault G., Pletcher D. (1991). Improvement to the equation for the steady state limiting currents at a microelectrode: EC’ processes (1st and 2nd order reactions). J. Electroanal. Chem. Interfacial Electrochem..

[17] Lavagnini I., Pastore P., Magno F. (1993). Digital simulation of steady state and non-steady state voltammetric responses for electrochemical reactions occurring at an inlaid microdisk electrode: Application to ECirr, EC’ and CE first-order reactions. J. Electroanal. Chem..

[18] Tutty O.R. (1994). Second-order kinetics for steady state EC0 reactions at a disc microelectrode. J. Electroanal. Chem..

[19] Molina A. (1998). Analytical solution corresponding to the i/t response to a multipotential step for a catalytic mechanism. J. Electroanal. Chem..

[20] Molina A., Serna C., Martinez-Ortiz F. (2000). Square wave voltammetry for a pseudo-first-order catalytic process at spherical electrodes. J. Electroanal. Chem..

[21] Mirceski V., Gulaboski R. (2001). Surface catalytic mechanism in square-wave voltammetry. Electroanalysis.

[22] Mirceski V., Gulaboski R. (2003). The surface catalytic mechanism: A comparative study with square-wave and staircase cyclic voltammetry. J. Solid State Electrochem..

[23] Compton R., Banks C.E. (2011). Understanding Voltammetry.

[24] Molina A., Gonzalez J., Laborda E., Wang Y., Compton R.G. (2011). Analytical theory of the catalytic mechanism in square wave voltammetry at disc electrodes. Chem. Chem. Phys..

[25] Ward K.R., Lawrence N.S., Hartshorne R.S., Compton R.G. (2011). Cyclic voltammetry of the EC’ mechanism at hemispherical particles and their arrays: The split wave. J. Phys. Chem. C.

[26] Calhoun R.L., Bard A.J. (2011). Study of the EC’ mechanism by scanning electrochemical microscopy (SECM). ECS Trans..

[27] Gulaboski R.L., Mihajlov L. (2011). Catalytic mechanism in successive two-step protein-film voltammetry—Theoretical study in square-wave voltammetry. Biophys. Chem..

[28] Gulaboski R., Mirceski V., Bogeski I., Hoth M. (2012). Protein film voltammetry: Electrochemical enzymatic spectroscopy: A review on recent progress. J. Solid State Electrochem..

[29] Yue D., Jia Y., Yao Y., Sun J., Jing Y. (2012). Structure and electrochemical behavior of ionic liquid analogue based on choline chloride and urea. Electrochim. Acta.

[30] Gulaboski R., Mirceski V. (2015). New aspects of the electrochemical-catalytic (EC’) mechanism in square-wave voltammetry. Electrochim. Acta.

[31] Vorotyntsev M.A., Konev D.V., Tolmachev Y.V. (2015). Electroreduction of halogen oxoanions via autocatalytic redox mediation by halide anions: Novel EC mechanism. Theory for stationary 1D regime. Electrochim. Acta.

[32] Vorotyntsev M.A., Antipov A.E. (2016). Reduction of bromate anion via autocatalytic redox-mediation by Br2/Br redox couple. Theory for stationary 1D regime. Effect of different Nernst layer thicknesses for reactants. J. Electroanal. Chem..

[33] Vorotyntsev M.A., Antipov A.E. (2017). Bromate electroreduction from acidic solution at rotating disc electrode. Theory of steady-state convective-diffusion transport. Electrochim. Acta.

[34] Vorotyntsev M.A., Volgin V.M., Davydov A.D. (2022). Halate electroreduction from acidic solution at rotating disc electrode. Theoretical study of the steady-state convective-migration-diffusion transport for comparable concentrations of halate ions and protons. Electrochim. Acta.

[35] Konev D.V., Antipov A.E., Petrov M.M., Shamraeva M.A., Vorotyntsev M.A. (2017). Surprising dependence of the current density of bromate electroreduction on the microelectrode radius as manifestation of the autocatalytic redox-cycle (EC″) reaction mechanism. Electrochem. Commun..

[36] Skrabal A., Schreiner H. (1934). Die Reduktionsgeschwindigkeit der Chlorsäure und Bromsäure. Monatshefte für Chemie und verwandte Teile anderer Wissenschaften.

[37] Taube H., Dodgen H. (1949). Applications of radioactive chlorine to the study of the mechanisms of reactions involving changes in the oxidation state of chlorine. J. Am. Chem. Soc..

[38] Hong C.C., Lenzi F., Rapson W.H. (1967). The Kinetics and mechanism of the chloride-chlorate reaction. Can. J. Chem. Eng..

[39] Lenzi F., Rapson W.H. (1968). Effets ioniques spécifiques sur le taux de formation du ClO2 par la réaction chlorure–chlorate. Can. J. Chem..

[40] Hoq M.F., Indu B., Ernst W.R. (1992). Kinetics and mechanism of the reaction of chlorous acid with chlorate in aqueous sulfuric acid. Ind. Eng. Chem. Res..

[41] Schmitz G. (1999). Kinetics and mechanism of the iodate–iodide reaction and other related reactions. Phys. Chem. Chem. Phys..

[42] Vogt H., Balej J., Bennett J.E., Wintzer P., Sheikh S.A., Gallone P., Vasudevan S., Pelin K., Ullmann F. (2010). Chlorine Oxides and Chlorine Oxygen Acids. Ullmann’s Encyclopedia of Industrial Chemistry.

[43] Sant’Anna R.T.P., Santos C.M.P., Silva G.P., Ferreira R.J.R., Oliveira A.P., Côrtes C.E.S., Faria R.B. (2012). Kinetics and mechanism of chlorate-chloride reaction. J. Braz. Chem. Soc..

[44] Lipsztajn M. (1988). Electrolytic Protection of Chlorine Dioxide. U.S. Patent.

[45] Tian M., Li Y.Y., Sun H.C., Yang L.J., Li Z.L. (2013). Preparation of chlorine dioxide by electrocatalytic reduction of sodium chlorate. Adv. Mater. Res..

[46] Arenas L.F., Walsh F.C., de León C.P. (2015). The importance of cell geometry and electrolyte properties to the cell potential of Zn-Ce hybrid flow batteries. J. Electrochem. Soc..

[47] Mussini T., Longhi P., Bard A.J., Parsons R., Jordan J. (1985). The Halogens. Bromine. Standard Potentials in Aqueous Solutions.

[48] Gomez-Gonzalez A., Ibanez J.G., Vasquez-Medrano R.C., Paramo-Garcia U., Zavala-Araiza D. (2009). Cathodic Production of ClO_2_ from NaClO_3_. J. Electrochem. Soc..

[49] Ni Y., Yin G. (1998). Disproportionation of chlorous acid at a strong acidity. Ind. Eng Chem. Res..

[50] Pichugov R.D., Konev D.V., Petrov M.M., Antipov A.E., Loktionov P.A., Abunaeva L.Z., Usenko A.A., Vorotyntsev M.A. (2020). Electrolyte Flow Field Variation: A Cell for Testing and Optimization of Membrane Electrode Assembly for Vanadium Redox Flow Batteries. ChemPlusChem.

[51] Ren X., Wang Y., Liu A., Zhang Z., Lv Q., Liu B. (2020). Current progress and performance improvement of Pt/C catalysts for fuel cells. J. Mater. Chem. A.

