# Supplementary material for: Hydrogen-Chlorate Electric Power Source: Feasibility of the Device, Discharge Characteristics and Modes of Operation
Source: Molecules. 2022 Sep 1;27(17):5638. doi: 10.3390/molecules27175638 (PMC9457794; doi:10.3390/molecules27175638)
Supplement: Supplementary file 1 [file molecules-27-05638-s001.zip › molecules-1848496-supplementary.pdf]

# Supplementary materials

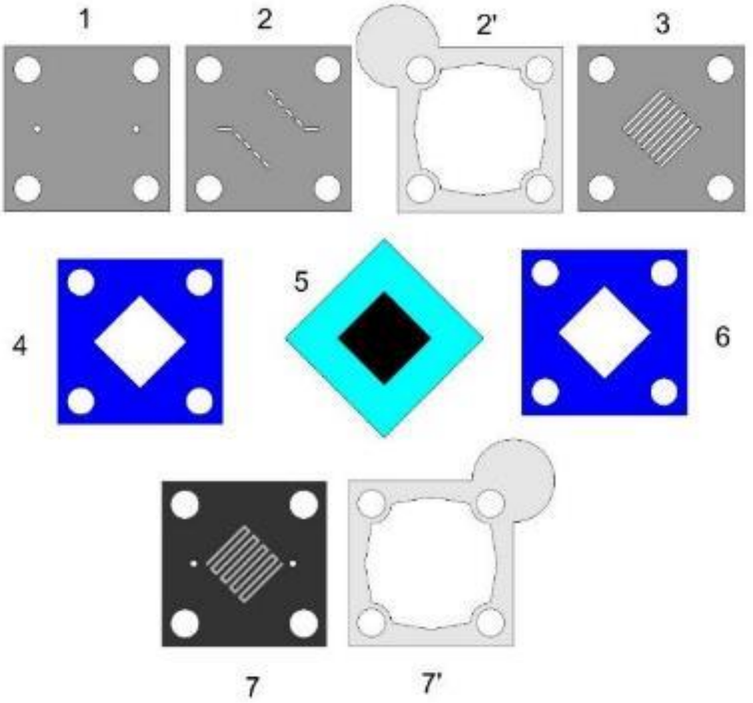


Figure S1. Design of the discharge cell (surface area: 2x2 $cm^2$): graphite foil with channels for supplying hydrogen 1,2,3; flow restricting frames (Teflon, Viton) 4,6, Membrane-Electrode Assembly 5 (electrodes are shown in black, membrane is shown in blue), glassy carbon plate with channel for catholyte flow supply and distribution 7; current collectors 2',7 (copper or nickel foil, 0.1 mm). Assembled from the bottom up in the order: 1-2-2'-3-4-5-6-7-7'. Hydrogen and catholyte supply lines are attached to end plates (not shown in the figure).

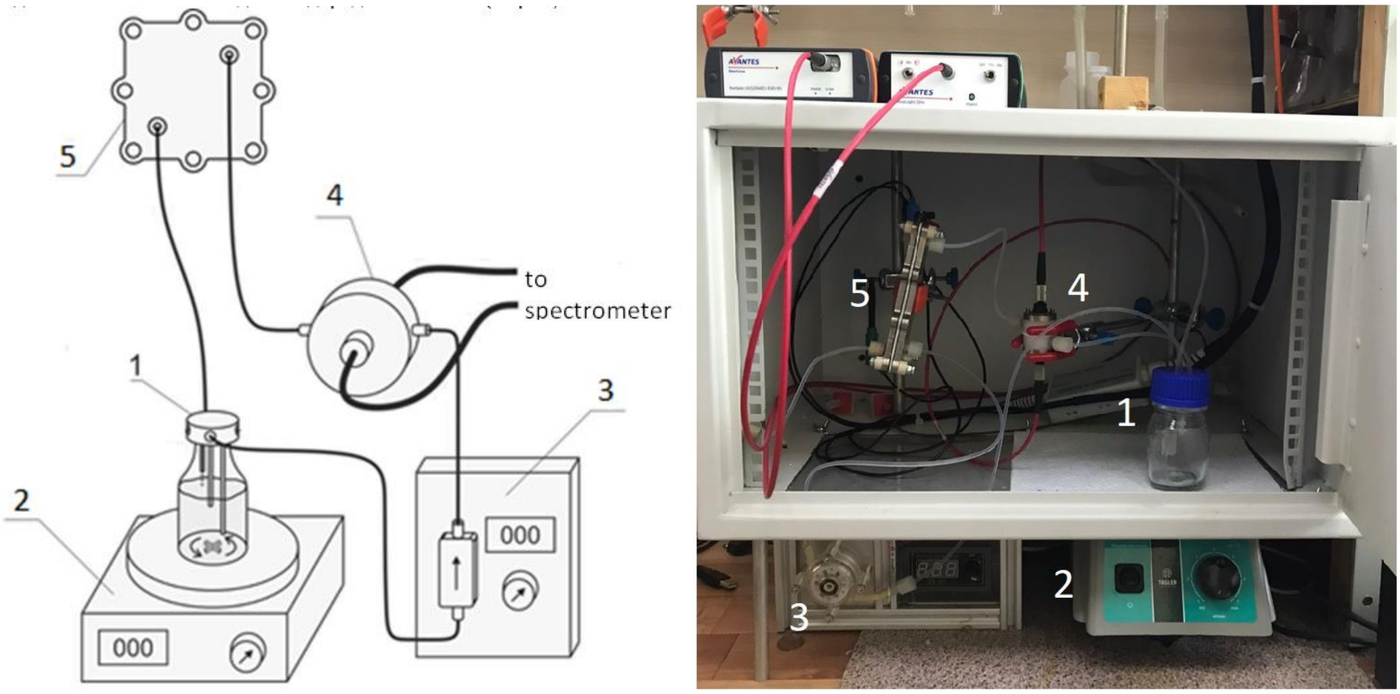


Figure S2. Scheme (left) and photo (right) of the experimental setup (cathode circuit) for testing the MEA of hydrogen-chlorate battery: tank with chlorate electrolyte 1; magnetic stirrer 2; pump 3; flow spectrophotometric cell with fiber optical cables 4; MEA cell 5.

To analyze the content of chloride ion in water seal installed on the hydrogen exit line from anode, a set of measurements was carried out. 10 ml aliquot of the total volume of water in hydraulic seal (40 ml) was placed in electrochemical cell, 1.32 ml of concentrated $H_2SO_4$ (98%) was added; thus, 2M $H_2SO_4$ solution of chloride ions was obtained. Then, the resulting solution was analyzed by square-wave voltammetry on compact platinum electrode (circular section of Pt wire; diameter: 1 mm; area: 0.785 $mm^2$) within the potential range from 0.6 to 1.5 V vs. Ag/AgCl reference electrode; sweep rate: 125 mV/s . Then, initial chloride concentration in solution was determined with the use of calibration curve (Fig. 3SM (b)) after taking into account the dilution with sulfuric acid.

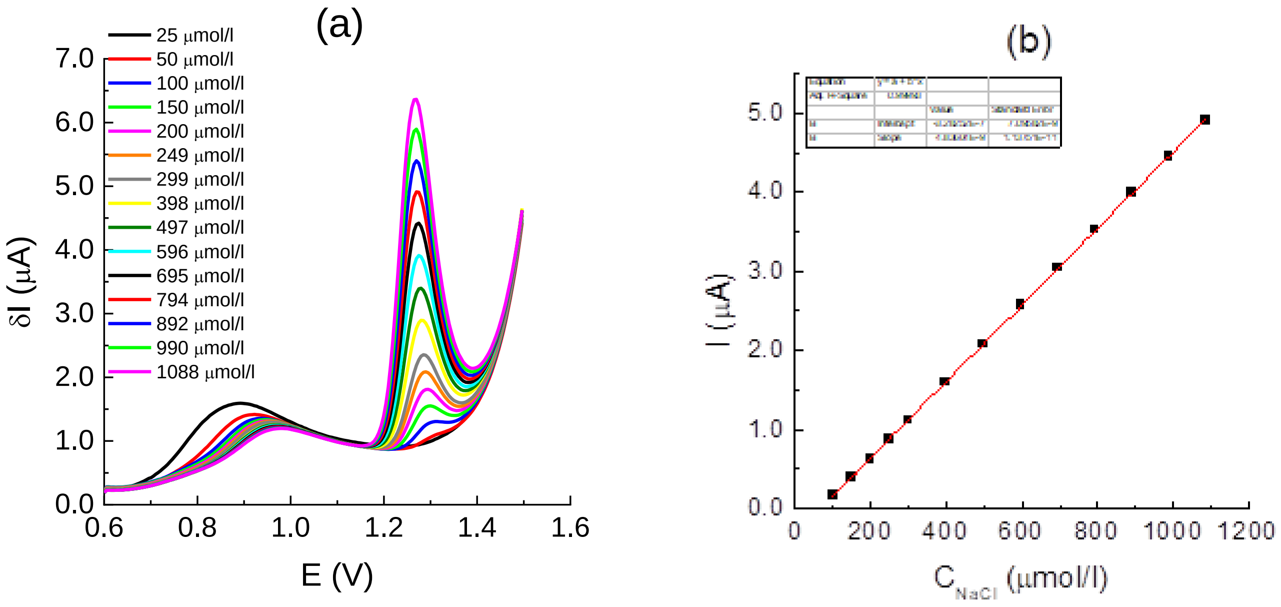


Figure S3. Square-wave voltammogram (a) and calibration curve in coordinates: peak current vs. chloride anion concentration (b) for 2M $H_2SO_4$ solution where black points correspond to data in Fig. 3SM (a). Potential step amplitude: 20mV; frequency: 25 Hz; step: 5 mV, measurement range: from 0.6 to 1.5 V vs. Ag/AgCl, $KCl_{sat}$ reference electrode
